# The underdog invader: Breeding system and colony genetic structure of the dark rover ant (*Brachymyrmex patagonicus* Mayr)

**DOI:** 10.1002/ece3.5917

**Published:** 2019-12-08

**Authors:** Pierre‐André Eyer, Elida M. Espinoza, Alexander J. Blumenfeld, Edward L. Vargo

**Affiliations:** ^1^ Department of Entomology Texas A&M University College Station TX USA; ^2^ EnviroFlight, LLC Yellow Springs OH USA

**Keywords:** ants, colony breeding system, invasive species, monogyne, multicolonial structure

## Abstract

Ants are among the most successful species at invading new environments. Their success undeniably comes from their various modes of reproduction and colony breeding structures, which influence their dispersal ability, reproductive potential, and foraging strategies. Almost all invasive ant species studied so far form supercolonies, a dense network of interconnected nests comprising numerous queens, without aggression toward non‐nestmates. This strategy results in invasive colonies that are able to grow extremely fast and large while avoiding intraspecific competition, allowing them to monopolize environmental resources and outcompete native species.

Here, we developed and used 10 microsatellite markers to investigate the population structure and breeding system of the dark rover ant *Brachymyrmex patagonicus* Mayr in its introduced range. We determined whether this species exhibits a supercolonial structure by assessing whether different nests belonged to the same genetic colony. We inferred its dispersal ability by investigating isolation by distance and estimated the numbers of queens per colonies and mating per queen through parent‐offspring inferences. We found that most of the colonies of *B. patagonicus* were comprised of a single nest, headed by a single queen. Each nest was distinct from one another, without isolation by distance, which suggests strong dispersal ability through nuptial flights. These features are commonly observed in noninvasive and native ant species, but they are surprising for a successful invasive ant, as they strongly differ from other invasive ants. Overall, we discuss how this seemingly unfavorable strategy for an invasive ant might favor the invasive success of the dark rover ant in the United States.

## Graphical abstract

Almost all invasive ant species studied so far form supercolonies, a dense network of interconnected nests comprising numerous queens, without aggression toward non‐nestmates. Here, we show that most of the colonies of invasive ant *Brachymyrmex patagonicus* were comprised of a single nest, headed by a single queen. Each nest was distinct from one another, without isolation by distance, which suggests strong dispersal ability through nuptial flights. These features are commonly observed in noninvasive and native ant species, but they are surprising for a successful invasive ant.

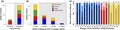

## 1 INTRODUCTION

Unraveling the factors underlying invasion success is a major goal of invasion biology. This is generally investigated at different stages of the invasion process: introduction, establishment, and expansion of exotic species in their invasive ranges. This question is particularly relevant for the over 200 ant species that have been reported as introduced (McGlynn, 1999; Suarez, McGlynn, & Tsutsui, 2010). Most introduced ants remain restricted to disturbed habitats in localized areas, rely on human‐mediated transport (so called, “tramp” species; Hölldobler & Wilson, 1990; Passera, 1994), and have little biological or economic impact. However, some introduced ants are considered invasive, becoming widespread from their initial introduction point, reaching high population densities and causing significant ecological and/or economic impacts (Holway, Lach, Suarez, Tsutsui, & Case, 2002; McGlynn, 1999). Ant species exhibit a broad diversity in their modes of reproduction and colony breeding structures, which influences their dispersal ability, reproductive potential, and foraging strategies. However, many invasive species share a common reproductive and colony breeding strategy that may facilitate their invasion success.

Numerous reproductive queens are commonly found within the nests of invasive ant species (i.e., polygyne), allowing them to increase colony growth and survival (Boomsma, Huszár, & Pedersen, 2014; Boulay, Arnan, Cerdá, & Retana, 2014; Passera & Keller, 1994). Polygyny increases colony survival, as the fate of colonies is not tied to the death of one queen. This breeding system also favors invasion success through the production and the allocation of a high number of workers to dominate resources (Tsutsui, Suarez, Holway, & Case, 2000). Polygyny is usually associated with the spread of colonies by budding, in which new nests are founded from a split of a mature nest (Boulay et al., 2014; Keller, 1991). During this process, daughter queens leave the natal nest on foot with a fraction of the worker force to establish new nests nearby (Cronin, Molet, Doums, Monnin, & Peeters, 2013). Colony budding may enhance successful expansion as each colony fragment contains both fertile queens and a substantial worker force, thus increasing its viability and reproduction (Chang, 1985; Hee, Holway, Suarez, & Case, 2000).

In addition, introduced populations of most invasive ant species are unicolonial with colonies comprised of several interconnected nests that exchange workers, queens, and brood. The development of these supercolonies arises through the loss of aggressive behavior toward non‐nestmates (Jackson, 2007; Steiner et al., 2007). This social structure favors invasion success, as the absence of conspecific competition enables invasive colonies to grow extremely large and monopolize environmental resources (Holway, Suarez, & Case, 1998; Tsutsui et al., 2000). Unicolonial populations therefore form dense networks of nests, each one housing numerous reproductive queens. Even though unicoloniality is a common trait in invasive social insects, both the size and number of supercolonies may differ among and within species, with a supercolony defined as the range of the ant where workers from distinct nests lack aggression toward one another. For example, the invasive population of the big‐headed ant *Pheidole megacephala* in northeastern Australia forms a vast supercolony covering up to 3,000 km (Fournier, Biseau, & Aron, 2009). A similar pattern is observed in the tawny crazy ant *Nylanderia fulva* in its invasive range in the southern US, where the population consists of a single supercolony spread across more than 2,000 km (Eyer, McDowell, et al., 2018). In contrast, the population of the yellow crazy ant *Anoplolepis gracilipes* comprises at least six supercolonies on the small island of Borneo (Drescher, Blüthgen, Schmitt, Bühler, & Feldhaar, 2010; Thomas, Becker, Abbott, & Feldhaar, 2010). In the Argentine ant *Linepithema humile*, the size and the number of supercolonies differ among its various invasive populations, which consist of one to five supercolonies ranging from 1 to 6,000 km long (Björkman‐Chiswell, Wilgenburg, Thomas, Swearer, & Elgar, 2008; Buczkowski, Vargo, & Silverman, 2004; Corin, Abbott, Ritchie, & Lester, 2007; Giraud, Pedersen, & Keller, 2002; Hirata, Hasegawa, Toita, & Higashi, 2008; Suhr, McKechnie, & O'Dowd, 2009; Suhr, O'Dowd, McKechnie, & Mackay, 2011; Thomas, Payne‐Makrisâ, Suarez, Tsutsui, & Holway, 2006; Vogel, Pedersen, Giraud, Krieger, & Keller, 2010). To date, the combination of polygyny, colony budding dispersal, and unicolonial structure has been described in many invasive ants species, including the most widespread and destructive ones (reviewed in Holway et al., 2002), such as *L. humile* (Giraud et al., 2002), *Lasius neglectus* (Cremer, Ugelvig, & Drijfhout, 2008), *Wasmannia auropunctata* (Fournier et al., 2005), *P. megacephala* (Fournier et al., 2009), *Monomorium pharaonis* (Buczkowski & Bennett, 2009; Schmidt, d'Ettorre, & Pedersen, 2010), *M. floricola* (Wetterer, 2010), *A. gracilipes* (Thomas et al., 2010), *N. fulva* (Eyer, McDowell, et al., 2018), *Tapinoma melanocephalum* (Zheng, Yang, Zeng, Vargo, & Xu, 2018), and the polygyne form of *Solenopsis invicta* (Fletcher, Blum, Whitt, & Temple, 1980). Interestingly, these strategies have also been reported in a lesser extend in the invasive ant *Brachyponera chinensis* (Eyer, Matsuura, et al., 2018), in the tramp ant species of the genus *Cardiocondyla* (Heinze, Cremer, Eckl, & Schrempf, 2006) and in introduced populations of the termite *Reticulitermes urbis* (Leniaud, Pichon, Uva, & Bagnères, 2009). Overall, these findings show that these features are common in invasive ant species, allowing them to efficiently expand, rapidly outcompete native species, and achieve local dominance in their new environment (Holway et al., 2002; Tsutsui et al., 2000).

The dark rover ant *B. patagonicus* Mayr is a very small ant (maximum 2.5 mm length) that has established in the southern US from its native range in Argentina and Paraguay (Quirán, Martínez, & Bachmann, 2004). First reported in 1976 (Wheeler & Wheeler, 1978), it has spread considerably in recent years across the southern US from Georgia to California (MacGown, Hill, & Deyrup, 2007; Martinez, 2010), moving northbound through Tennessee and North Carolina (Hill, 2017). In its native range, this species inhabits a broad diversity of environments, including naturally disturbed habitats (Calcaterra, Cabrera, & Briano, 2016). *Brachymyrmex patagonicus* is a significant nuisance pest infesting buildings (MacGown et al., 2007), but, in contrast with other invasive ant species in the United States, this species has not received great attention, most likely due to its tiny size, or its lack of biting, stinging, and mechanical transmission of disease (MacGown et al., 2007). Although the social structure of this species has never been explicitly studied, nests are usually small and contain hundreds of workers, sometimes found in extremely close proximity (a few centimeters from each other), that display considerable mutual tolerance (MacGown et al., 2007). These findings, together with infestation sizes that can be extremely large, may suggest that this species forms supercolonies made of interconnected nests without aggression between nestmate workers.

In this study, we investigated the population genetic structure and breeding system of the dark rover ant *B. patagonicus* in its introduced range. We tested whether this species displays the combination of polygyny, colony budding dispersal, and unicolonial structure commonly observed in other invasive ant species. We first assessed patterns of genetic diversity within and across populations to determine whether this species exhibits a unicolonial structure or whether each nest represents a genetically distinct colony. We evaluated population structure and isolation by distance among populations to infer the dispersal abilities of this species in the United States. Within each nest, we estimated the number of queens and the number of matings per queen. Finally, we discussed the mechanisms underlying the invasion success of the dark rover ant in the United States.

## 2 MATERIALS AND METHODS

**Figure 1:**
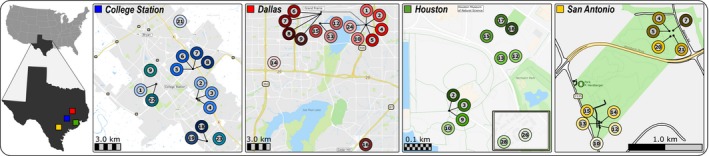
Sampling locations of the 50 nests of *Brachymyrmex patagonicus* sampled in the four localities in Texas

A total of 50 nests of *B. patagonicus* were collected in four localities in Texas over 2017 and 2018 (Figure 1). Fourteen nests were collected in Bryan–College Station (BCS), 15 in Dallas–Fort Worth (DFW), 11 in Houston (HOU), and 10 in San Antonio (SAN). In order to determine whether nests belonged to the same polydomous colony, nests were collected as close as 1 m apart. In addition, new queens and males were found for seven nests (see detailed sampling in Table S1 and Figure S2). All workers and sexual offspring were directly stored in pure ethanol at −20°C until DNA extraction.

The DNA of 4–26 workers, as well as all the new (alate) queens present and up to 12 males per nest, was extracted following a modified Gentra PureGene protocol (Gentra Systems, Inc.). A total of 821 workers from 50 nests (mean ± *SD *= 16.4 ± 4.9), 25 new queens from three nests (mean ± *SD *= 8.3 ± 5.8), and 39 males from six nests (mean ± *SD* = 6.5 ± 4.1) were analyzed (Table S1).

Twenty‐three new microsatellite markers (*Bpa1* to *Bpa23*; Table S2) were developed for *B. patagonicus* running a pool of 10 individuals originating from the College Station, TX population through one lane of Illumina MiSeq. Reads were trimmed of low‐quality bases (*Q* score < 25) and adapters using Trimmomatic (Bolger, Lohse, & Usadel, 2014). Trimmed sequences were assembled using ABySS v.1.9.0 (Simpson et al., 2009). Among the 751,412 contigs analyzed, 122,304 of them contained penta‐, tetra‐, tri‐, or dinucleotide microsatellite repeat motifs, as identified by Msatcommander v. 0.8.2.0 (Faircloth, 2008). A pair of primers was designed for each of the 23 loci selected (13 tri‐ and 10 tetra‐nucleotide repeat loci) using the online Primer3 software (http://www.simgene.com/Primer3). Fifteen microsatellite markers were successfully amplified on a subset of twenty *B. patagonicus* workers. Two monomorphic microsatellite markers (*Bpa6* and *Bpa21*) were later discarded, as well as three microsatellite markers (*Bpa2*, *Bpa17*, and *Bpa22*) due to inconsistent amplification. No evidence of null alleles or linkage disequilibrium for any of the remaining loci was observed after Bonferroni corrections for multiple testing. The final microsatellite dataset comprised of 10 microsatellite markers (Table S2). These microsatellites were genotyped using the M13‐tailed primer method (Boutin‐Ganache, Raposo, Raymond, & Deschepper, 2001), where M13 tails are 5′‐fluorescently labeled with 6‐FAM, VIC, PET, or NED dyes to facilitate multiplexing. DNA amplifications were performed in a volume of 10 µl including 0.25–1.0 U of MyTaq™ HS DNA polymerase (Bioline), 2 µl of MyTaq™ 5x reaction buffer (Bioline), 0.08 µl of each primers, and 1.25 µl of the DNA template. PCR was carried out using a Bio‐Rad thermocycler T100 (Bio‐Rad). PCR products were visualized on an ABI 3500 genetic analyzer and sized against LIZ500 internal standard (Applied Biosystems). Allele labeling was performed using Geneious software v.9.1 (Kearse et al., 2012).

A fragment of the COI mitochondrial gene was sequenced using the *Jerry* and *Pat* primer pair (Simon et al., 1994) for one individual per nest (*N* = 50). PCR products were purified with EXOSAP‐it PCR purification kit (Applied Biosystems) and sequenced with the ABI BigDye Terminator v.3.1 Cycle Sequencing Kit (Applied Biosystems) on an ABI 3500 capillary sequencer (Applied Biosystems). Base calling and sequence reconciliation were performed using CodonCode Aligner (CodonCode Corporation). Only two haplotypes were found for all the 50 samples analyzed across the four localities (see Section 3), which preclude the use of this marker for any robust analyses of population structure or colony structure aiming at inferring the number of matrilines within colonies.

### 2.1 Population and colony structure

Allele frequencies, observed and expected heterozygosity, and *F*‐statistics were assessed using FSTAT (Goudet, 1995). Population and colony structure were determined for the four localities using only the worker dataset. Essentially, we determined whether all populations of *B. patagonicus* consist of a single large supercolony, several supercolonies of smaller size, or whether every nest consists of a separate colony. We answered this question at the overall population scale, then within each of the four localities. At both scales, we used three different methods. First, genotypic frequencies at all nests were compared using a log‐likelihood (G)‐based test of differentiation using GENEPOP ON THE WEB (Rousset, 2008), in order to test whether different pairs of sampling nests belonged to the same genetic entity (i.e., colony). A Bonferroni correction was applied to account for multiple comparisons of all nest pairs. Significance was determined using a Fisher's combined probability test. Second, population and colony clustering were visualized by plotting individuals on a principal component analysis (PCA) using *Adegenet R* package (Jombart, 2008). Third, the most likely number of genetic clusters (*K*) was estimated using Bayesian clustering implemented in STRUCTURE v.2.3.4 (Pritchard, Stephens, & Donnelly, 2000), to assess whether distinct nests cluster under a unique colony entity. A clustering simulation was first run for the overall invasive population, and then followed by separate simulations for each locality. The simulations were run with values of *K* from 1 to the total number of nests encountered (overall = 50, then BCS = 14, DFW = 15, HOU = 11 and SAN = 10) and repeated 20 times for each *K*‐value. The analyses were performed using an admixture and a correlated‐allele frequencies model without a priori hypothesis based on geographic locations. Each run included a 5 × 10^4^ burn‐in period followed by 1 × 10^5^ iterations of the MCMC. The most likely number of groupings was evaluated using the Δ*K* method (Evanno, Regnaut, & Goudet, 2005) implemented in Structure Harvester v.0.6.8 (Earl & vonHoldt, 2012).

Finally, we investigated whether genetic differentiation between nests increased with geographic distances by plotting [*F*_ST_/(1 − *F*_ST_)] coefficients between pairs of nests against the natural log (*ln*) of their geographic distance (Slatkin, 1993). We tested the significance of the correlation using the Mantel tests implemented in GENEPOP ON THE WEB (Rousset, 2008). We also investigated pattern of distribution of the genetic diversity at different hierarchical scales (i.e., among localities, among nests within localities, and within nests) using an analysis of molecular variance (AMOVA) implemented in Arlequin (Excoffier & Lischer, 2010).

### 2.2 Breeding system

For each nest (all of them consisting of distinct colonies, see Section 3 below), the number of queens was directly reported from field observations. This number was later genetically confirmed, or not, using two complementary approaches. First, 4–26 workers per colony (mean ± *SD* = 16.4 ± 4.9) were genotyped to infer whether they could all be assigned to a single queen (carrying one of the two alleles of the mother queen at each microsatellite marker studied). Polygyny was deduced when more than 1 worker per colony could not be unambiguously assigned to a single queen. Second, when available, 2–12 males per colony were genotyped to confirm the number and the genotype of the queen(s) (males are haploid, and carry only maternal alleles as they are produced through arrhenotokous parthenogenesis).

For monogyne colonies, queen‐mating frequency was determined from mother‐offspring analyses inferring the number of males and their genotypes from the workers' genotypes. Each worker was assigned to a given patriline with the maximum‐likelihood method implemented in the software COLONY v.1.2 (Wang, 2004). To ensure confidence in the number of males inferred, the probability of nondetection of an extra male carrying the exact same genotype at all loci studied was calculated according to the equation of Boomsma and Ratnieks (1996):

$$P_{\text{non}\;-\;\text{detection}}=\Pi_{j}\sum_{i}f_{\mathrm{ij}}^{2}$$

where *f_ij_* is the frequency of the allele *i* at the locus *j*.

Relatedness coefficients (*r*) among nestmate workers were estimated using the program COANCESTRY v.1.0 (Wang, 2011), according to the algorithm described by Queller and Goodnight (1989). Relatedness coefficients were weighted equally, and standard errors (*SE*) were obtained by jackknifing over colonies.

As thelytokous parthenogenesis for the production of new queens seems to be particularly frequent in invasive ant species (e.g., Fournier et al., 2005; Pearcy, Goodisman, & Keller, 2011), we assessed whether new queens were asexually produced while sexual reproduction is conserved for worker production in *B. patagonicus*. Using the three colonies in which new queens were sampled (mean ± *SD* = 8.3 ± 5.8), we estimated asexual production of new queens by comparing whether they carry only the two maternal alleles at all markers studied. Additionally, we estimated relatedness coefficients among new queens within colonies.

## 3 RESULTS

### 3.1 Population and colony structure

**Figure 2:**
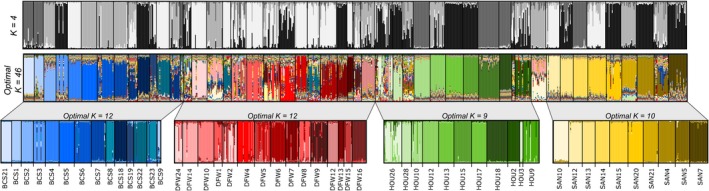
Graphical representation of STRUCTURE results determining the number of genetic groups in the entire population in Texas (*n* = 821; *N* = 50 nests). Each group is characterized by a color, and each individual is represented by a vertical bar according to its probability to belong to each group. Distinct simulations were later run for the four localities, separately

**Figure 3:**
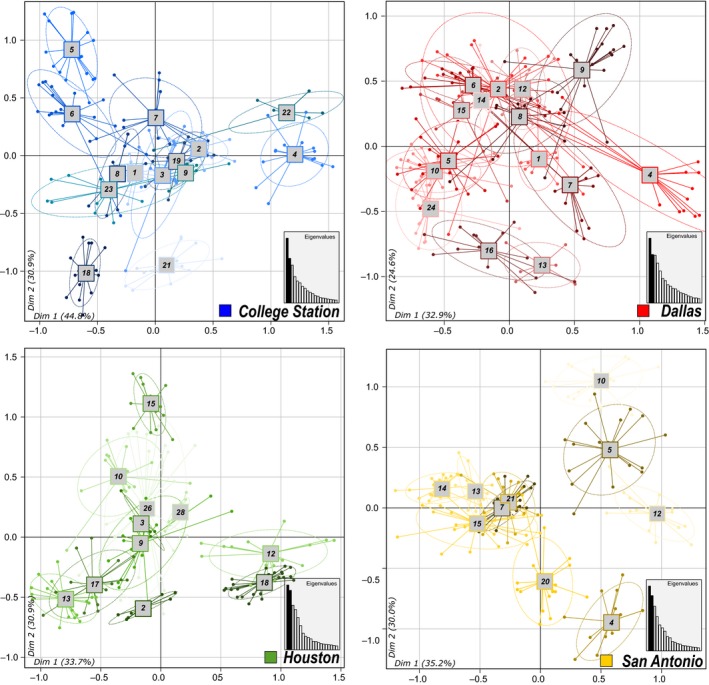
Clustering of nests within the four localities studied using principal component analyses of the microsatellite markers. Clustering of nests in the overall population is given in Figure S1

Only two haplotypes were found on the 633 bp fragment of the COI mitochondrial marker for all the 50 samples analyzed across the four localities. The two haplotypes differ by seven nucleotides. The final microsatellite dataset contains 10 polymorphic microsatellite markers, with allele numbers ranging from 2 to 13 (mean ± *SD* = 6.4 ± 4.3). Significant genetic differentiation among nests was found in the overall introduced population, with *F*_ST_ = 0.329. This conclusion was also noticeable within each locality, as significant population structure was observed among nests within localities (*F*_ST_ = 0.37, 0.26, 0.36, and 0.33 for BCS, DFW, HOU, and SAN, respectively). Consistent with these results, the overall G test, as well as each pairwise G test within localities, revealed that nearly all of the nests could be genetically differentiated (97.5% of the pairs of nests tested). In addition, the STRUCTURE analysis revealed the occurrence of several genetic clusters, with optimal *K* = 46, mostly corresponding to the different nests sampled (Figure 2). However, it failed to segregate the four localities into homogeneous clusters when the entire population was analyzed. This indicates a strong genetic differentiation between nests, but a weak population structure between localities. Similar results were found when each locality was analyzed separately, with nests clearly distinct from one another (Figure 2). The segregation of the genetically different nests was also evident using the principal component analyses, as nestmate workers grouped together, while the different nests scattered along the axes, at both locality and population scales (Figure 3, PCA on the overall population is given in Figure S1). Overall, these results suggest that the invasive *B. patagonicus* population does not consist of either a single supercolony, or of different smaller supercolonies within each locality. Rather, this population of *B. patagonicus* is composed of distinct, monodomous colonies.

**Figure 4:**
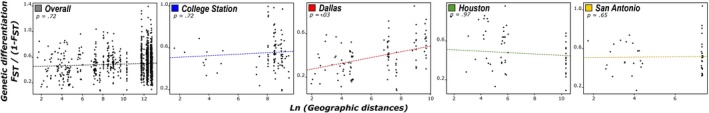
Correlations between genetic differentiation between nests and geographic distances (isolation by distance) using microsatellite markers. The analysis was first performed for the overall population and then performed for each locality, separately

The analysis of molecular variance on the microsatellite markers gave similar results; only 1.2% of the genetic diversity observed in the invasive population of *B. patagonicus* was distributed among the four localities, 30.8% among the nests within localities, while 67.9% within the nests (Table 1). No relationship between pairwise *F*_ST_ and geographic distances was observed in most localities, except for the DFW population (Figure 4). This absence of significant correlation is unlikely to be an artifact of the small sampling distances within localities, as this pattern was also observed at the overall population scale (Figure 4). These results are consistent with the fact that STRUCTURE strongly segregates distinct nests, but fails to separate the four localities. They confirm the strong genetic differentiation between nests but the lack of population structure between localities. Overall, these results suggest a substantial dispersal ability of *B. patagonicus* that allows for homogenization of genetic diversity despite a large geographic range.

**Table 1:** Analysis of molecular variance (AMOVA) at different hierarchical levels for populations of *Brachymyrmex patagonicus*

Source of variation	Sum of squares	Variance components	Percentage variation
Among localities	111.09	0.03	1.25
Among nests within localities	1,077.90	0.70	30.80
Within nests	2,358.03	1.55	67.95
Total	3,547.02	2.29	

### 3.2 Breeding system

**Figure 5:**
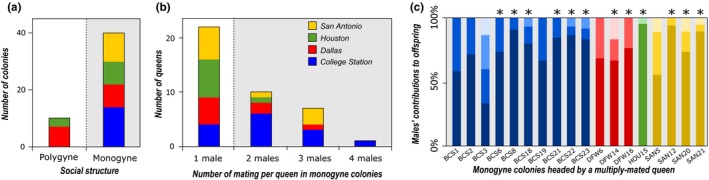
Number of monogyne and polygyne colonies of *B. patagonicus* uncovered in each locality (a). Number of mating per queen for each monogyne colony (b). Offspring contribution of each patriline uncovered in the different polyandrous colonies (c)

A single queen was found in 19 out of 50 of the nests sampled. The presence of a single reproductive queen per nest was genetically confirmed for all of them, as well as for 21 nests for which no queen was sampled, as all worker genotypes could be unambiguously assigned to the queen. In addition, the reproduction of a single queen was also supported by male genotypes in the six colonies for which males were available. In total, 40 colonies were found to be monogyne (Figure 5a). The 10 remaining colonies were found polygyne by our genetic analyses; however, no polygyne colony was directly observed from the field. For monogyne colonies, mother‐offspring comparisons indicated that nearly half the queens were singly mated (22 out of the 50 queens analyzed). Eleven queens were mated with 2 males, 7 with 3 males and only one with 4 males, no more than 4 matings per queen was inferred (*M*_p_ ± *SD *= 2.50 ± 0.61; Figure 5b). The probability that additional males sharing the same alleles at all loci went undetected was very low (*p*_nondetection_ = .00032). Interestingly, a high and significant reproductive bias was found in almost all polyandrous queens with the queens' mates contributing unequally to the production of workers. As a consequence, the mean effective number of matings per queen was low (*M*_e,p_ ± *SD* = 1.79 ± 0.76; Figure 5c). The relatedness among nestmate workers was therefore quite high, with *R*_W‐W_ = 0.63 (±*SD* = 0.17, ranging from 0.16 to 0.84). Notably, the average relatedness within polygyne colonies was still fairly high, with *R*_W‐W_ = 0.43 (±*SD* = 0.16, ranging from 0.16 to 0.64), indicating the presence of only a few reproductive queens. Interestingly, the inbreeding coefficient was strongly negative (overall *F*_IS_ = −0.429). We found similar values within each locality (*F*_IS_ = −0.48, −0.29, −0.475, and −0.526 in BCS, DFW, HOU, and SAN, respectively). As a result, workers exhibit a high level of heterozygosity, significantly higher than that expected under random mating. This finding may suggest that queens mated with highly unrelated males or simply reflect the nonindependence of genotypes from a single family.

In the three colonies for which new queens were available, the new queens carried both maternal and paternal alleles at each marker analyzed, suggesting that they were produced through classical sexual reproduction. In addition, the relatedness among queens did not differ from the relatedness among nestmate workers (*R*_Q‐Q_ = 0.70, 0.72 and 0.70; *R*_W‐W_ = 0.71, 0.77, and 0.66, for colonies HOU13, HOU18, and SAN5, respectively).

## 4 DISCUSSION

This study provides several new insights into the life history of the dark rover ant *B. patagonicus* in its invasive range in the United States. Its breeding system and colony structure strongly contrast with those uncovered in many other invasive ant species, as *B. patagonicus* exhibits a multicolonial and monodomous colony structure. Each colony comprised a single nest, clearly different from one another, even those separated by a couple of meters. No association between genetic differentiation and geographic distance was uncovered, suggesting that this species displays strong dispersal ability, most likely spreading through nuptial flights. Further, 80% of the colonies of *B. patagonicus* are headed by a single queen, which is mated, half of the time, by a single male. These features are commonly observed in noninvasive and native ant species, but they are surprising for a successful invasive ant such as *B. patagonicus*. These characteristics strongly differ from other highly dominant invasive species, as they are usually highly polygyne and disperse by budding, and form supercolonies that may sometimes spread across the whole invasive range of the species.

*Brachymyrmex patagonicus* is an emerging pest that was introduced into the United States in the 1970s. Less than five decades later, this species is now commonly found in at least 12 states in the United States, from North Carolina to California (Hill, 2017; MacGown et al., 2007; Martinez, 2010). This shows that *B. pata*gonicus has been highly successful in its introduction, establishment and spread within the United States. Despite our genetic findings await behavioral data, they suggest that *B. patagonicus* exhibits a multicolonial structure where nests are most often headed by a single queen. This strategy is most likely to be similar to that of the populations in its native range in South America, suggesting that its invasion success cannot be attributed to a shift in its breeding system and colony structure. Many invasive ant species experience a transition from multicoloniality to unicoloniality while invading a new environment. In their native ranges, they usually display monodomous colonies, or small polydomous colonies (Errard, Delabie, Jourdan, & Hefetz, 2005; Eyer, McDowell, et al., 2018; Heller, 2004; Ross, Vargo, & Keller, 1996; Suarez, Tsutsui, Holway, & Case, 1999). These colonies are genetically distinct from one another and colony members exhibit aggressive behaviors toward non‐nestmates. On the other hand, those same ant species exhibit unicolonial populations consisting of a single, or a few, vast supercolonies in their invasive ranges. These supercolonies are made of multiple nests, each containing up to hundreds of reproductive queens.

The development of supercolonies generally observed in the introduced range of most invasive species may be explained by the absence of competitors, thereby allowing introduced colonies to grow and spread widely. This is supported by the pre‐existence of polydomous colonies in the native range, albeit several orders of magnitude smaller than those observed in the introduced range (Errard et al., 2005; Giraud et al., 2002; Heller, 2004; Le Breton, Delabie, Chazeau, Dejean, & Jourdan, 2004; Pedersen, Krieger, Vogel, Giraud, & Keller, 2006; Suarez et al., 1999; Tsutsui & Case, 2001). Other scenarios suggest a transition from multicoloniality in the native range to unicoloniality in the invasive range, arising from an absence of aggression between non‐nestmate workers (Giraud et al., 2002). The absence of non‐nestmate antagonism could have been selected for as aggressive behaviors are costly in densely populated introduced ranges (Chapuisat, Bernasconi, Hoehn, & Reuter, 2005; Holway, 1999; Holway et al., 1998; Katzerke, Neumann, Pirk, Bliss, & Moritz, 2006), or is a result of the loss of nestmate recognition. The founder effect during an invasion could have reduced the genetic diversity at the loci influencing the cuticular hydrocarbons used to discriminate non‐nestmates (Beye, Hasselmann, Fondrk, Page, & Omholt, 2003; Lockey, 1991; Pirk, Neumann, Moritz, & Pamilo, 2001; Reeve, 1989; Tsutsui et al., 2000). This results in homogenized colony templates, leading to a worker's inability to discriminate between nestmates and non‐nestmates (Tsutsui, Suarez, & Grosberg, 2003; Tsutsui et al., 2000; Vásquez, Schal, & Silverman, 2008).

The *Brachymyrmex* genus contains some of the smallest ants in the family Formicidae, with a total of 44 described species (Bolton, Alpert, & Ward, 2007), workers measuring <2 mm in length. Because *B. patagonicus* is a tiny ant, colonies can fit into small spaces and can therefore be easily transported in wood materials (MacGown et al., 2007). Potentially, this feature may have led to multiple introductions of this species in the United States, thus reducing the extent of the bottleneck experienced. The absence of genetic reduction in its invasive range may have preserved the ability of workers to discriminate non‐nestmates, thereby maintaining genetic differentiation between nests (Thomas et al., 2010). In rare cases, multiple introductions have resulted in the introduced range being as genetically diverse as the native range, and sometimes even more diverse if the different introductions came from distinct native populations (Dlugosch & Parker, 2008). In *B. patagonicus*, no data are available on the genetic diversity of the native range, making the comparison between native and introduced ranges and testing for multiple introductions currently impossible. However, the finding of weak mitochondrial diversity in the invasive range may suggest that this species actually experienced a genetic bottleneck from a single or few introduction events.

To the best of our knowledge, the red imported fire ant *Solenopsis invicta* is the only invasive ant species that displays a monogyne social structure in its invasive range. However, this species actually exhibits two social structures, a polygyne form and a monogyne one. Both forms have successfully invaded the Southern US (Ross & Fletcher, 1985; Ross, Krieger, & Shoemaker, 2003), suggesting that their social structure is not the driving factor behind their invasion success. Several features of the monogyne form of *S. invicta* have been compared to weed proliferations and are suggested to enhance its invasiveness (Tschinkel, 1987). Colonies of the monogyne form exhibit a high reproductive rate and strong dispersal ability, as they invest in a high number of reproductives that disperse through nuptial flights (Ross & Keller, 1995a, 1995b). This allows monogyne colonies to reach unconnected patches and spread faster than polygyne colonies that disperse through budding (Resasco et al., 2014). In colonies of the monogyne form, the production of reproductives occurs early, half of them producing new sexuals by the end of their first year. In addition, they exhibit efficient colonization and achieve rapid colony growth during the first stage of colony foundation (Tschinkel, 1987), as colonies are founded by multiple foundress queens (i.e., pleiometric foundation) (Tschinkel & Howard, 1983). This feature boosts the initial production of workers, thereby increasing the survival rate of founding colonies (Tschinkel & Howard, 1983), while monogyny is restored at the end of the claustral period. Monogyne colonies show strong aggression toward non‐nestmate workers, whereas aggressive behaviors between polygyne colonies are reduced (Morel, Meer, & Lofgren, 1990). Our results suggest that *B. patagonicus* colonies possess many of the same weedy characteristics as the monogyne form of *S. invicta*, including a strong ability to disperse through nuptial flights. Furthermore, the social form of *S. invicta* colonies is genetically determined by the heterozygosity of queens and workers at a specific genomic region (*Gp‐9* supergene; Krieger & Ross, 2002; Ross & Keller, 1998; Wang et al., 2013). Homozygous colonies are monogyne, while heterozygous ones are polygyne. Therefore, the relative abundance of each social form is linked to the allelic diversity at the supergene region within the population. In *B. patagonicus*, most colonies (80%) are monogyne, whereas polygyne colonies have been uncovered in only two of the four localities. The genetic influence upon social structure and the relative investment in reproductives of *B. patagonicus* colonies therefore warrant future study.

Although social Hymenoptera (i.e, ants, bees, and wasps) are among the most successful invasive species, they are particularly sensitive to inbreeding depression that usually follows the founding event during introduction (Zayed & Packer, 2005). These species display a single‐locus complementary sex determination under which males arise from hemizygous, haploid eggs, while females (i.e., queens and workers) develop from heterozygous eggs (Beye et al., 2003; Heimpel & Boer, 2008; Schmieder, Colinet, & Poirié, 2012). Homozygous diploid individuals at the sex locus develop into diploid sterile males in place of females (Jones & Brown, 2014; Ross, Vargo, Keller, & Trager, 1993); hence, they represent a severe cost for colonies and a risk of extinction for the population (van Wilgenburg, Driessen, & Beukeboom, 2006; Zayed & Packer, 2005). As a consequence, bottlenecks are particularly detrimental for social Hymenoptera, as they rely on genetic diversity at the sex‐locus to produce the female castes.

Several mating strategies have been described in invasive social Hymenoptera species that allow them to cope with the reduction of genetic diversity within introduced populations. In some invasive populations of the ant species *W. auropunctata*, *Vollenhovia emeryi*, *A. gracilipes* and *Paratrechina longicornis*, new queens are clones of their mothers while sons are clones of their fathers, thereby creating distinct female and male lineages (Drescher, Biüthgen, & Feldhaar, 2007; Fournier et al., 2005; Ohkawara, Nakayama, Satoh, Trindl, & Jr, 2006; Pearcy et al., 2011). Since workers arise from the sexual reproduction of both lineages, they are 100% heterozygous. In these species, a single queen may invade and establish a new population. The queen's daughter queens and sons can therefore mate together without inducing inbreeding depression in their offspring. This unorthodox strategy circumvents the costs of inbreeding within bottlenecked populations, therefore acting as a pre‐adaptive trait to invasion (Pearcy et al., 2011). In the Asian needle ant *Brachyponera chinensis*, the genetic diversity and level of heterozygosity within introduced colonies are similar to those observed in the native range, despite the severe bottleneck experienced by the introduced population (Eyer, Matsuura, et al., 2018). In this species, inbreeding does not result from the founder effect, but rather due to sibmating pre‐existing in the native range. Generations of sibmating in native populations may have reduced inbreeding depression through the purifying selection of deleterious alleles, and thus lowered the cost of invasion (Eyer, Matsuura, et al., 2018). In the invasive bee *Apis cerana*, polyandry may reduce the extent of the bottleneck by artificially increasing the number of migrants into the introduced range, thus increasing diversity at the sex‐locus. Through their storage of sperm, polyandrous founding queens bring a reservoir of genetic diversity from the native population (Ding, Xu, Oldroyd, & Gloag, 2017). This diversity is later transmitted directly through the production of new queens by the founding queen (i.e., founding queens' sons do not carry paternal (i.e., sperm) alleles). Remarkably, the large fraction of males produced through worker reproduction allows for an additional indirect transmission of genetic diversity at the sex‐locus (Gloag et al., 2019). In *B. patagonicus*, we did not find any diploid males (*N* = 39); yet, this species did not exhibit any of the strategies mentioned above. Queens did not use asexual reproduction for the production of new queens, while all males analyzed carried only maternal alleles, indicating an absence of clonal reproduction by males and a lack of worker reproduction. Most queens of *B. patagonicus* were mated with a single male, ruling out the possibility that polyandry may have reduced the bottleneck of this species during its introduction. Nevertheless, we found a strong negative inbreeding coefficient in all populations analyzed for all microsatellite markers, revealing that queens mated with highly unrelated males. This outbreeding strategy may reduce the probability of mating with a male carrying the exact same allele at the sex‐locus, thus avoiding the costly production of diploid males.

In most invasive ant species, the sustainability of unicolonial structure seems paradoxical, as the numerous queens and the loss of colony boundaries reduce nestmate relatedness to zero, greatly decreasing their indirect fitness (Hamilton, 1964; Helanterä, Strassmann, Carrillo, & Queller, 2009). In the absence of worker fitness benefits, selfish behaviors are expected to challenge social cohesion within the colonies (Keller, 1997). An absence of positive relatedness was observed within supercolonies of distinct invasive species (e.g.,* S. invicta* (Ross et al., 1996)*; L. humile* (Tsutsui et al., 2000); *L. neglectus* (Cremer et al., 2008); *P. megacephala* (Fournier et al., 2009); *N. fulva* (Eyer, McDowell, et al., 2018)). However, when several genetically distinct supercolonies coexist within introduced populations, selection for worker altruism is still expected to occur at supercolony boundaries, as colony members are more related to each other than to members of other supercolonies. In *A. gracilipes*, several supercolonies occupy the small island of Borneo (Thomas et al., 2010). As a result, contacts between workers from distinct supercolonies are frequent, and workers of a given supercolony are more likely to compete against workers from a distinct one. The relatedness coefficients within supercolonies are therefore quite high in this species (Drescher et al., 2007; Thomas et al., 2010). In *B. patagonicus*, each nest was clearly distinct from one another. Due to this multicolonial structure and the low number of reproductives per colony, the relatedness observed in this species is also quite high (*R*_W‐W_ = 0.63). The breeding system observed in *B. patagonicus* consequently stands in stark contrast with those reported in most other invasive ant species.

Unicolonial and highly polygyne invasive populations are efficient at producing a high number of workers to monopolize resources (Boomsma et al., 2014; Boulay et al., 2014), allowing them to outcompete native ant species (Tsutsui et al., 2000). However, the multicolonial and monogyne structure of the introduced *B. patagonicus* population probably does not provide this species with highly competitive abilities. Rather, its subordinate behavior may play a major role in its successful invasion of the southeastern US (MacGown et al., 2007). *B. patagonicus* is described as opportunistic, displaying submissive behavior toward larger and more aggressive ants (Amatta, Calcaterra, & Giannoni, 2018; Carval, Cotte, Resmond, Perrin, & Tixier, 2016; Leal et al., 2017). Both in its native and introduced ranges, this species has been found nesting side‐by‐side with highly dominant and aggressive ant species, such as the fire ant *S. invicta*,* Dorymyrmex bureni*, *P. moerens*, and *P. obscurithorax* (Calcaterra et al., 2016; MacGown et al., 2007), sometimes even within the same wooden debris*.* In the United States, the fire ants' presence and abundance have no effect on the abundance of *B. patagonicus* (MacGown et al., 2007). In addition, *B. patagonicus* has never been reported to displace other ant species, suggesting that they can tolerate and co‐occur with several ant species. Interestingly, this is in sharp contrast with the monogyne form of *S. invicta* (i.e., the other monogyne invasive ant species), which is highly aggressive toward a large number of native and introduced ant species (Tschinkel, 1988), displacing most of them (Gotelli & Arnett, 2000; Wojcik, 1994). For these reasons, the success of *B. patagonicus* does not seem to be attributed to superior competitive and aggressive abilities. Instead, it stems from their capacity to inhabit a variety of natural and disturbed habitats allowing them to colonize fragmented anthropic environments (Calcaterra et al., 2016), and from their ability to thrive in the presence of other native and invasive ant species, even highly aggressive ones.

## 5 CONCLUSION

The breeding system and social structure we described in *B. patagonicus* are common in ants. The occurrence of a distinct colony within each single nest, headed by a unique, singly mated queen is observed in most ant species, and embodies the ancestral structure of social Hymenoptera (Hughes, Oldroyd, Beekman, & Ratnieks, 2008). Yet, these findings represent a very unusual strategy for an invasive ant species. Almost all invasive ant species form supercolonies, each one comprised of numerous nests containing up to thousands of reproductive queens. The occurrence of this unicolonial structure in almost all invasive ant species seemingly indicates that this type of social system provides a competitive superiority for invasive ants. However, this study reveals that an invasive ant species displaying a very classical breeding system might be as successful as unicolonial populations. These results highlight the complexity of the mechanisms underlying invasion success, suggesting that several biotic or abiotic factors may alter the course of an invasion, thereby greatly affecting the success of an invasive species—even those with an “underdog” reproductive strategy.

## CONFLICT OF INTEREST

None declared.

## AUTHOR CONTRIBUTIONS

ELV and EE designed the study. EE collected samples. EE developed the microsatellites. PAE and EE performed the genetic analyses. PAE and AJB analyzed the data. PAE wrote the paper with contributions of AJB, ELV, and EE.

## Data Availability

The datasets generated during the current study are available in the Dryad repository, doi‐numbers: https://doi.org/10.5061/dryad.5nq1pf7
