# Supplementary figures and images for: The underdog invader: Breeding system and colony genetic structure of the dark rover ant (Brachymyrmex patagonicus Mayr)
Source: Ecol Evol. 2019 Dec 8;10(1):493–505. doi: 10.1002/ece3.5917 (PMC6972842; doi:10.1002/ece3.5917)

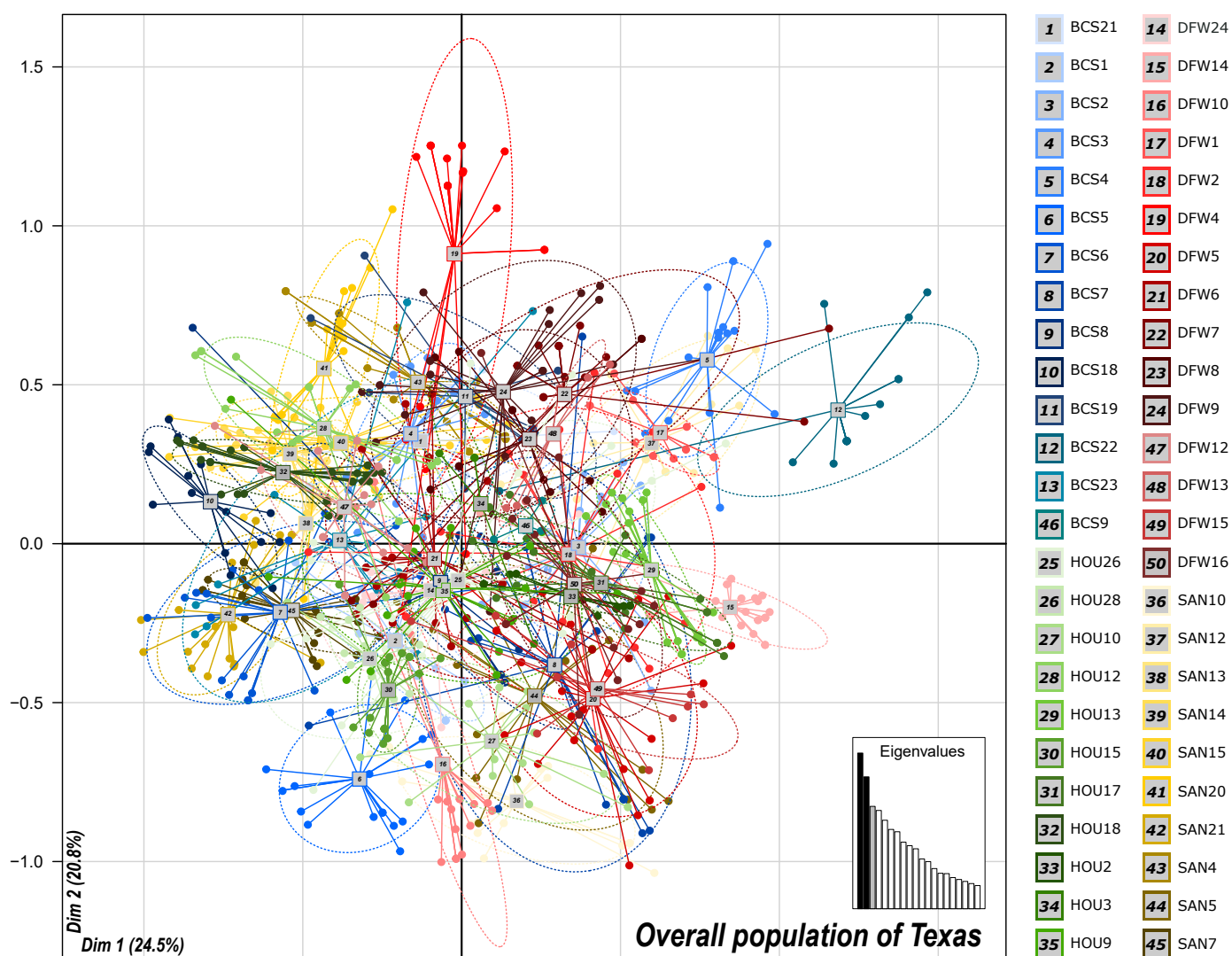

**Figure S1:** Clustering of the colonies within the overall population based on PCA results

Supplement: Supplementary file 1 [file ECE3-10-493-s001.pdf]

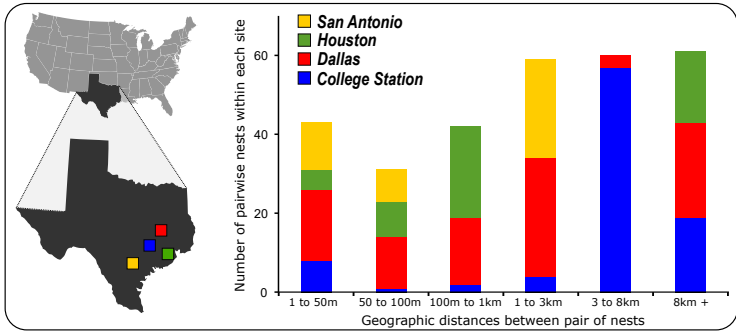

**Figure S2:** number of pairwise nests in each category of geographic distances for each site

Supplement: Supplementary file 2 [file ECE3-10-493-s002.pdf]
