# Supplementary material for: The underdog invader: Breeding system and colony genetic structure of the dark rover ant (Brachymyrmex patagonicus Mayr)
Source: Ecol Evol. 2019 Dec 8;10(1):493–505. doi: 10.1002/ece3.5917 (PMC6972842; doi:10.1002/ece3.5917)
Supplement: Supplementary file 3 [file ECE3-10-493-s003.pdf]

Table S1: Localities, number of queen, number of mating per queen, number of samples analyzed and geographic coordinates for every colony of *Brachymyrmex patagonicus* sampled.

Locality	Colony	Number of queens field	Number of queens genetic	Number of mating per queen	# samples genotyped	GPS coordinates	
Bryan / College Station	BCS1	1	1	2	10W	30,62184	-96,36023
	BCS2	-	1	2	15W	30,60790	-96,31270
	BCS3	-	1	4	15W	30,60782	-96,31276
	BCS4	1	1	1	17W	30,60756	-96,31304
	BCS5	-	1	1	19W	30,63886	-96,31659
	BCS6	-	1	2	21W	30,63910	-96,31566
	BCS7	-	1	1	18W	30,63952	-96,31551
	BCS8	1	1	2	11W	30,63924	-96,31522
	BCS9	-	1	1	4W	30,63710	-96,36900
	BCS18	1	1	3	15W	30,56296	-96,29897
	BCS19	-	1	2	9W	30,56297	-96,29892
	BCS21	-	1	2	13W	30,68801	-96,33218
	BCS22	-	1	3	16W	30,62211	-96,36016
	BCS23	1	1	3	13W	30,56397	-96,28478
Dallas/Fort Worth	DFW1	-	1	1	10W	32,74189	-96,98916
	DFW2	-	***P***	-	19W	32,74184	-96,98876
	DFW4	-	***P***	-	21W	32,74171	-96,98902
	DFW5	-	***P***	-	20W	32,74134	-96,98896
	DFW6	-	1	2	19W	32,74074	-96,98907
	DFW7	-	***P***	-	16W	32,74076	-96,98920
	DFW8	-	***P***	-	15W	32,74070	-96,98913
	DFW9	-	***P***	-	21W	32,74082	-96,98915
	DFW10	-	***P***	-	21W	32,74186	-96,98937
	DFW12	1	1	1	13W	32,72863	-96,99146
	DFW13	-	1	1	7W	32,72874	-96,99155
	DFW14	1	1	3	10W	32,68500	-97,05194
	DFW15	-	1	1	10W	32,72030	-96,99630
	DFW16	-	1	2	17W	32,58606	-96,93272
	DFW24	-	1	1	19W	32,74178	-96,98919
Houston	HOU2	-	1	1	15W	29,70754	-95,38844
	HOU3	1	1	1	4W	29,70765	-95,38477
	HOU9	-	***P***	-	20W	29,71811	-95,38965
	HOU10	-	***P***	-	14W	29,71808	-95,38947
	HOU12	1	1	1	21W	29,72063	-95,38683
	HOU13	1	1	1	18W - 15Q - 2M	29,72059	-95,38729
	HOU15	1	1	2	22W	29,72117	-95,38760
	HOU17	1	1	1	19W - 10M	29,72134	-95,38694
	HOU18	1	1	1	26W - 5Q - 12M	29,72140	-95,38695
	HOU26	-	***P***	-	22W	29,55047	-95,13225
	HOU28	-	1	1	13W	29,55011	-95,13379
San Antonio	SAN4	1	1	1	21W	29,56167	-98,52052
	SAN5	1	1	3	18W - 5Q	29,56163	-98,52048
	SAN7	-	1	1	23W - 2M	29,56166	-98,51999
	SAN10	-	1	1	19W	29,55001	-98,53041
	SAN12	-	1	2	17W	29,55018	-98,53042
	SAN13	1	1	1	16W	29,55038	-98,53041
	SAN14	1	1	1	18W - 7M	29,55042	-98,53028
	SAN15	-	1	1	23W - 6M	29,55052	-98,53044
	SAN20	1	1	3	19W	29,56100	-98,52033
	SAN21	1	1	3	19W	29,56105	-98,51999

*P = polygyne colony

*W = worker
*Q = new queen
*M = male
