# Supplementary material for: The underdog invader: Breeding system and colony genetic structure of the dark rover ant (Brachymyrmex patagonicus Mayr)
Source: Ecol Evol. 2019 Dec 8;10(1):493–505. doi: 10.1002/ece3.5917 (PMC6972842; doi:10.1002/ece3.5917)
Supplement: Supplementary file 4 [file ECE3-10-493-s004.pdf]

The Underdog Invader: Breeding system and colony genetic structure of the dark rover ant *Brachymyrmex patagonicus*

Eyer et al.

Table S2: DNA extraction protocol, primer sequences, motifs, number of repeats, amplification protocol and number of alleles for each microsatellite marker studied

name	Motif and number of repeats	Product Size	Primer Sequences Primer F	Primer R	Annealing T°C	Post-PCR & PCR Multiplexing	Number of Alleles
Bpa1	(ATT)^6	/	CACGACGTTGTAAAACGACCTTCAATCTCAATAGTGCGCC	GGGAATTTGCGGTCGGTTC	/	/	/
Bpa2	(AAT)^18	/	CACGACGTTGTAAAACGACGACAGACGCCAGACTGCAC	CGCGCGAGCCAAATTATCG	/	/	/
Bpa3	(ATCT)^11	/	CACGACGTTGTAAAACGACTCGAGTGCATATTTAGCGCC	GCGCTCTCGCGATTTCATAC	/	/	/
Bpa4	(GCT)^5	193-196	CACGACGTTGTAAAACGACAGCACTGAGCCAACTAGCG	TGCTCCTGCGATTCGGTC	55	B2	2
Bpa5	(AAGG)^6	205-209	CACGACGTTGTAAAACGACCGCGTGCATCCTTCAATCG	AGGCACTTTGAGGGCTCTG	55	B1	2
Bpa6	(ACG)^5	200	CACGACGTTGTAAAACGACGGTGGTGCTTCGAAATCGG	TGCCACAGAGCCGTAGAAC	55	A2	1
Bpa7	(CCT)^5	207-216	CACGACGTTGTAAAACGACTCGGCTGGATGGATTTCCC	CGAACGGGATGCTCCTTTG	50,3	C1	4
Bpa8	(CTT)^6	216-222	CACGACGTTGTAAAACGACTTTGAACGAATCGACCTCAAG	TTGGATTCCGAGCGTGAAC	50,3	C3	3
Bpa9	(AACG)^7	216-259	CACGACGTTGTAAAACGACTGTCGAATGACGGGATCGG	TAGGGCACGGATGGTGAAC	50,3	C2	13
Bpa10	(CAGT)^5	/	CACGACGTTGTAAAACGACAGTTGGTTTGACCGGTAAGTG	ACAACTGGATTGTTGCGCC	/	/	/
Bpa11	(ATC)^6	/	CACGACGTTGTAAAACGACGTAAGATCGGCGCTTTAGGG	CCTCAAAGGTGGCCTAACC	/	/	/
Bpa12	(AGC)^5	254-267	CACGACGTTGTAAAACGACAACACAACCTGCGTTCAAG	TCGTTTGTGCTACGCGTTC	55	B1	3
Bpa13	(CGT)^5	246-259	CACGACGTTGTAAAACGACACACGTTACAAGTGTATACCGAC	GTACGCCCGGTAAATTCGC	55	B2	5
Bpa14	(ACG)^8	279-312	CACGACGTTGTAAAACGACGCACGCCGACTTAATTTGC	AGAACCGATCAACCCGGAG	55	A2	10
Bpa15	(ACAT)^6	/	CACGACGTTGTAAAACGACCTCGTCCCATACGGCCAG	CAAACAAAGCCAGCGCC	/	/	/
Bpa16	(CTT)^11	287-308	CACGACGTTGTAAAACGACTCGCGATATTCCGCCTGAC	GACTCGCGTCAGAATTCCC	55	A1	12
Bpa17	(ATCG)^5	/	CACGACGTTGTAAAACGACCGTTATTCGCTCAGATCGGC	TCGCTCGAGATCGTTTCCC	/	/	/
Bpa18	(ACC)^5	/	CACGACGTTGTAAAACGACGTGCCAAACATGGGAGGTC	GCAGGCGCCGATGTATAAC	/	/	/
Bpa19	(ATGT)^6	/	CACGACGTTGTAAAACGACGTGTAGCGTTCACAAGCCC	TTCAAACGATTGCAGGCCG	/	/	/
Bpa20	(CTT)^5	/	CACGACGTTGTAAAACGACAGGGATCTCTGACTTACGCC	GTTGCCCGCAAAGCAATAC	/	/	/
Bpa21	(AATC)^7	336	CACGACGTTGTAAAACGACAGGGAAACAAGACAAACGGG	CAGGCGCATCCTTGCAATC	50,3	C2	1
Bpa22	(ATTT)^8	/	CACGACGTTGTAAAACGACAGGAATTTGCATAAGACGCAATC	GAAACTGTACGTATTCTTCCGC	/	/	/
Bpa23	(GAGT)^10	372-405	CACGACGTTGTAAAACGACCTCTAATCGCGCCGTATAGC	TTCACTCGCGCCTACCAAG	55	A1	10

/ indicates microsatellite markers that did not amplify

DNA EXTRACTION

Whole ants were separately removed from storage ethanol, air-dried, and crushed with disinfected pestles inside a 1.7-mL tube with 100 µL nuclei lysis solution (Promega, Madison, WI, USA) and 1 µL proteinase K (20 mg/mL) (Bioline, Memphis, TN, USA), followed by centrifugation at 4000 rpm for 30 seconds and an incubation at 55 ºC for 3 hours. Sample tubes were then incubated at -20 ºC for 30 minutes and 35 µL 8 M ammonium acetate (pH 8) was added to precipitate proteins, followed by centrifugation at 10000 rpm for 7 minutes. Sample tubes were removed from the centrifuge and the supernatant was transferred into a new 1.7-mL tube containing 100 µL cold isopropanol and 1 µL glycogen (8 µg/µL) (Thermo Fisher Scientific, Waltham, MA, USA), followed by a brief vortex and a 1-hour incubation at -20 ºC. Sample tubes were centrifuged at 10000 rpm for 15 minutes and isopropanol was poured out; 400 µL cold, 70% ethanol was added into each sample tube and was followed by centrifugation at 10000 rpm for 10 minutes. Ethanol was poured out carefully to leave only precipitated pellet in the sample tube and excess ethanol was evaporated by placing open sample tubes in vacufuge for 14 minutes. The vacufuge was not set to spin and the duration within depended on the ethanol volume remaining in the sample tube. Finally, the DNA was eluted in 15 µL TE buffer and incubated overnight at room temperature before storing at -20 ºC for subsequent genetic analyses.

The Underdog Invader: Breeding system and colony genetic structure of the dark rover ant *Brachymyrmex patagonicus*

MICROSATELLITE ANALYSIS

1.25 µl of the extracted DNA was amplified by PCR (30 cycles) at 10 microsatellite loci in 7 mixes (A1 to C3) using a Bio-Rad thermocycler T100 (Bio-Rad, Pleasanton, CA). The 7 PCR mixes were then combined into 3 Post-PCR mixes (A, B, C). The amplified products were separated on ABI 3500 capillary sequencer (Applied Biosystems, Foster City, CA, USA) and sized against LIZ 500 sizing standards (BioVentures, Murfreesboro, TN, USA). Controls for genotyping errors due to null alleles were analysed following the Expectation Maximization algorithm of Dempster et al. (1977) implemented in the FREE NA software (Chapuis & Estoup 2007). Additional tests of heterozygote deficiency and estimation of linkage disequilibrium were performed in GENEPOP on the Web (Rousset 2000).

Chapuis M-P, Estoup A. 2007. *Mol Biol Evol.* **24:** 621-631.
Rousset. 2000. *J Evol Biol*. **13:** 58-62.
Dempster AP, Laird NM, Rubin DB. 1977. *J R Stat Soc B*. **39**:1–38.
